# Quantitation of Arsenic Species in Urine for Exposure Assessment Studies

**DOI:** 10.6028/jres.093.059

**Published:** 1988-06-01

**Authors:** David A. Kalman

**Affiliations:** Department of Environmental Health, University of Washington, Seattle, WA 98195

## 1. Overview

Accuracy in trace level clinical or biomedical analyses is of increasing importance as population-based surveillance is more widely used to address issues of nutritional requirements, environmental exposures, and epidemiological outcomes. Among the uses of such surveillance, evaluation of community exposures from a local arsenic pollution source is one example. Urinary biological monitoring is a desirable tool used in conjunction with measurement of environmental levels of arsenic in order to permit assessment of total exposures and to provide a basis for consideration of the relative importance of different routes of exposure and of age-related behaviors in influencing exposure. Urinary arsenic concentrations are indicative of recent (previous 1–2 days) exposures to arsenic. When total urinary arsenic is measured, total arsenic intake is assessed. When exposures to inorganic arsenic (in the forms of oxides or salts of trivalent or pentavalent arsenic) are of interest, determination of urinary arsenic species comprising the major metabolites of inorganic arsenic exposures is most appropriate [1]. A recent two-year study of community exposures to environmental inorganic arsenic pollution utilized urinary arsenic speciation analysis to assess exposures. In these analyses, part-per-billion sensitivity was achieved with a mean precision of less than 12% coefficient of variation, and with excellent interlaboratory comparability.

## 2. Assay Method

**Figure 1:**
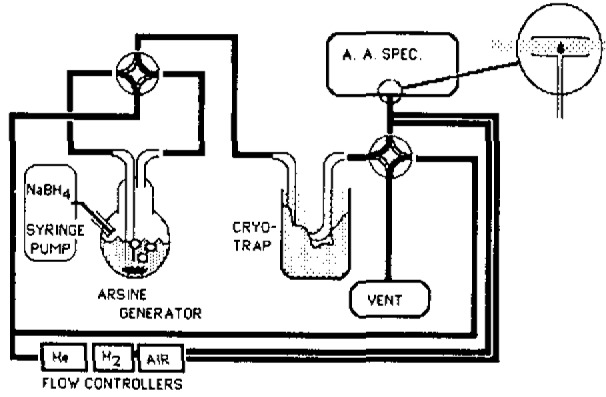
Analytical system schematic: Arsenic speciation assay.

The method utilizes a chemical reactor with addition of sodium borohydride to convert reducible arsenic compounds to the corresponding arsines and helium sparging to remove and concentrate arsines on a separation column maintained at cryogenic temperatures during trapping [2]. Following collection of all volatile arsenic, a temperature program is applied to the column and the eluted arsine species are detected by atomic absorption spectrometry using a microburner combustion cell [3]. Figure 1 shows a schematic of the analytical system. In order to increase sample throughput, two separate sample inlet trains were connected to the microburner cell using a 4-port glass valve. Arsine generation and cryogenic concentration could then occur on one inlet side while desorption and analysis was occurring on the other. Overall analysis time was thereby reduced to less than 15 minutes per sample, with up to 80 analyses being accomplished in a two-shift day. Analog signal output from the AA was integrated and concentrations computed using an electronic integrator. Direct serial interfacing to a MacIntosh-Plus microcomputer permitted us to download assay parameters and to transfer integrated results for processing in an electronic spreadsheet.

**Figure 2:**
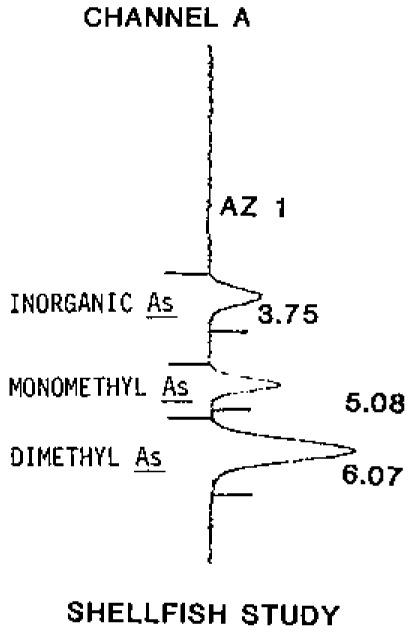
Typical analysis output.

The analytical sensitivity of the method is very high, due principally to the efficiency of the detection system. The method mass detection limit was 1 ng per compound, or 1 ppb for a 1 mL urine sample. Sample volumes of up to 100 mL were permitted by the capacity of the reactor used. A typical output chart is shown in figure 2.

## 3. Achieving Accuracy

### 3.1 Quality Control Procedures for the Assay

Initial method validation was accomplished using pooled urine obtained from arsenic-exposed smelter workers. Three pools (high, ca. 25 ppb; medium, ca. 15 ppb; and low, ca. 5 ppb arsenic) were provided to four laboratories routinely determining arsenic in urine samples. Each lab assayed the samples using its own standards and procedures, which ranged from calorimetric assay of total arsenic to speciation assay by methods essentially identical to ours. Of the five labs providing data, four showed good agreement (standard deviation of 3.6 ppb in high pool and 2.2 ppb in low pool), with the total arsenic assay slightly higher than the speciation assays. To maintain constant performance throughout the study, a benchmark sample was *prepared using* the mid-level urine pool frozen in 1500 aliquots. This sample was assayed daily for each inlet side at the beginning and end of each set of 7 samples. Control charts with 2-sigma limits were maintained daily. The daily analysis routine consisted of blanks, calibrants, benchmark samples, and replicate sample assays. Control samples in all accounted for approximately 26% of the analysis load.

### 3.2 Quality Assurance Procedures for the Project

Elements of the quality assurance effort were: the development of a QA manual for the assay and project; external audits by a technical team provided by EPA, Region X, EPA Las Vegas and its contractors, EPA RTP, and the Centers for Disease Control; and extralaboratory performance samples. The QA manual documented the SOP and subsequent modifications, set control limits and corrective actions required for out-of-control situations, and defined data validation criteria. Extralaboratory performance samples were provided quarterly by the EPA CLP QA office. In addition, blind resubmission of randomly-selected field samples and blind interlaboratory comparison samples shared with CDC were used to evaluate laboratory bias.

### 3.3 Assay Performance

Out-of-control events occurred approximately 6 times over the 14 months of intensive laboratory analysis. These events were typically related to failing source lamps, column degradation, or gas leaks. Blind sample resubmissions showed the same variation as within-lab reanalyses. All EPA performance audit samples were within control limits and were within 5 ppb of the reference value. Table 1 presents the summarized intra-laboratory QC results for the study.

## 4. Biomonitoring Results and Discussion

**Figure 3:**
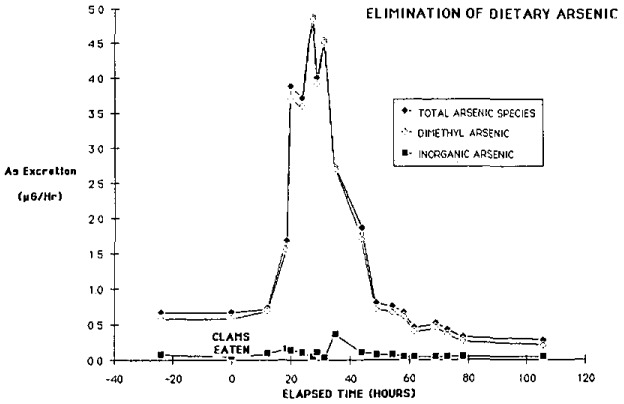
Time profile of excretion of urinary arsenic species following consumption of claws.

The completed data set for this study consisted of slightly more than 9000 urine sample assay results plus several thousand environmental measurements including arsenic in soil, ambient air particulate, personal air particulate, water and locally-raised foods, house dust, road dust, surface dust from playgrounds and schoolrooms, hand loadings, and hair. Statistical path analysis of this data set using several models showed that:

— For this population, environmental arsenic variation explained only about 25% of urinary arsenic variation, with age, sex, diet, and urinary creatinine covariation explaining another 25%. Unexplained variation could be divided as follows: within individuals (26%), between individuals within households (15%), and between households (8%).
— The most significant environmental compartments influencing urinary arsenic were: indoor and outdoor air concentration (for entire population), and soil arsenic (for ages 0–6 and 7–13 only); *p* <0.01 for each of these measures.
— Estimation of inhaled dose (based on personal air arsenic concentration and literature values for ventilation rate and retention of particulate) and comparison to mean arsenic daily intake (estimated by applying a steady-state pharmacokinetic model to those individuals whose urine values varied less than 25% between successive sampling days) showed that less than 15% of the arsenic intake could be contributed by inhalation, under reasonable worst-case assumptions.
— Dietary influences related to seafood consumption were not excluded by the use of speciation analysis. This was demonstrated both statistically and from feeding experiments, where some types of seafood (notably shellfish) produced significant short-term increases in urinary inorganic and methylated arsenic, as illustrated in figure 3.

## 5. Conclusions

We have demonstrated the feasibility of making large numbers of speciation measurements for urinary arsenic over a 14-month period, with good control of analytical performance and accuracy. Such measurements can permit the application of statistical tools to discern patterns of exposures leading to new insights regarding environmental pathways, target populations, and behavioral aspects of exposures. Needed developments to further this aim are reference materials with bioincorporated analytes, better procedures for normalizing urinary concentrations, and more complete pharmacokinetic characterization of arsenic for diverse age, sex, and exposure variables.

## Figures and Tables

**Table 1:** Quality control summary, AA/arsine speciation assay

*Samples assayed*	
Number of study samples	3728
Number of batches	375
Type of samples	water, urine, handwash
*QC summary*	
Blank analyses	
# required	424
# reported	424
# elevated blanks	none > LQL
Control samples	
# required	852
# reported	1186
mean “recovery” (% ref. value)	92.6±12.1
# outliers	128
definition of outliers	1 species not within 2−*σ* control limit
Replicate samples	
# required	249
# reported	251
mean precision	6.5% CV
# outliers	15 (6%)
definition of outliers	>25% CV and >2 ppb diff.
